# Simple Instrumental and Visual Tests for Nonlaboratory Environmental Control

**DOI:** 10.1155/2016/1270629

**Published:** 2016-05-09

**Authors:** L. P. Eksperiandova, S. V. Khimchenko, N. A. Stepanenko, I. B. Shcherbakov

**Affiliations:** SSI “Institute for Single Crystals”, National Academy of Sciences of Ukraine, Department of Analytical Chemistry of Functional Materials and Environment, Nauky Avenue, No. 60, Kharkiv 60001, Ukraine

## Abstract

Proposed are simple and available techniques that can be used for rapid and reliable environmental control specifically of natural water by means of instrumental and visual tests in outdoor conditions. Developed are the chemical colorimetric modes for fast detection of socially dangerous trace impurities in water such as Co(II), Pd(II), and Rh(III) as well as NO_2_^−^-ions and Fe(III) serving as model impurities. Application of portable digital devices and scanner allows estimating the color coordinates and increasing the accuracy and sensitivity of the tests. The combination of complex formation with preconcentration of colored complexes replaces the sensitive but time-consuming and capricious kinetic method that is usually used for this purpose at the more convenient and reliable colorimetric method. As the test tools, the following ones are worked out: polyurethane foam tablets with sorbed colored complexes, the two-layer paper sandwich packaged in slide adapter and saturated by reagents, and polyethylene terephthalate blister with dried reagents. Fast analysis of polyurethane foam tablets is realized using a pocket digital *RGB*-colorimeter or portable photometer. Express analysis of two-layer paper sandwich or polyethylene terephthalate blister is realized by visual and instrumental tests. The metrological characteristics of the developed visual and instrumental express analysis techniques are estimated.

## 1. Introduction

It is well known that ions of heavy metals, in particular, Co(II), Pd(II), and Rh(III), and especially their radioactive isotopes which are the products of uranium and plutonium fission [1], are socially dangerous pollutants. As a result of leakage of radioactive substances at nuclear accidents at power stations Fukushima (Japan) or Chernobyl (Ukraine), fission fragments of nuclear fuel get into the air, soil, and ocean [2], accumulate in living organisms, and enter the body [3, 4]. Therefore, the development of simple and express tests for online environmental monitoring is very important. Visual and instrumental express tests are the most suitable techniques for solving this problem. Digital image colorimetry is a relatively new and rapidly developing technique. It is most suitable, very promising, and highly informative for such application. The use of the tests is especially reasonable at screening the analyzed sample for fast “yes-no” response on the presence of the sought-for impurity.

Most of the reactions for detection of trace Co(II), Pd(II), and Rh(III) impurities are kinetic ones. As a rule, they are based on oxidation of azo compounds by strong oxidants accompanied with the color appearance or disappearance and catalyzed by traces of these elements. For example, the cobalt kinetic determination techniques are reported including oxidation of indigo carmine by H_2_O_2_ [5] and oxidation of pyrocatechin by H_2_O_2_ in the basic medium [6], as well as the sensitive and selective spectrophotometric method for Rh(III) determination based on its catalytic effect on the oxidation of Nile blue by periodate at pH 9.0 [7]. Despite the fact that the kinetic methods are characterized by high sensitivity, their widespread use is difficult due to their complicacy and capriciousness. Therefore, analysts try to use complexing reactions of the element to be determined and a suitable ligand which gives colored compounds. In particular, highly selective and sensitive method of spectrophotometric determination of trace amounts of Pd(II) based on the formation of a ternary complex Pd(II) with I^−^-ions and rhodamine 6G [8] has been described, as well as the method of sorption Co(II) on polyurethane foam (PUF) in the form of thiocyanate complexes [9].

Analytical reactions for visual or instrumental test analysis are carried out on a suitable solid carrier. By the extent of color change after the interaction with the solution (water) to be analyzed, one can judge about the concentration of the element to be determined. In most cases, carrying out a reaction proceeding on a solid carrier is connected with sorption of the reaction products by this solid carrier. The data concerning the solid carriers and their effectiveness for test application is given in the reviews [10, 11]. The analysis of the results reported in more than 200 publications during 15 years from the viewpoint of the frequency of the use of solid carrier for extraction from solutions or determination of heavy metals allows building the following succession: active carbon > graphite > silica gel > polymer sorbents > cellulose (paper) > fiber sorbents > metal oxides and hydroxides > naphthalene > zeolites. Based on these literature data, the authors have found the paper and PUF carriers to be the most convenient tools for such tests.

The aim of this study was to develop simple easy-to-use test tools to be applied for the known analytical reactions based on the change or appearance/disappearance of the analyte color due to the change of the determined element concentration, as well as to study the application of portable photometers for confident instrumental environmental monitoring under outdoor conditions. Such instrumental fast tests should make it possible to eliminate the negative effect of subjective factors on the analysis results.

## 2. Material and Methods

### 2.1. Reagents

For all the solutions, bidistilled and deionized water and reagents of chemically pure grade were used. The standard solutions (Fluka, Switzerland) of 1.00 g L^−1^ of Co(II) and Fe(III) in 0.001 mol dm^−3^ HNO_3_ as well as Rh(III) and Pd(II) solutions of 1.00 g L^−1^ in 5% HCl were used as initial solutions. All the work solutions were prepared by successive dilution of the initial ones with HNO_3_ (Merck, Germany) of the same concentration. NaNO_2_ (0.30 g L^−1^, Merck, Germany) solution was prepared by dissolving the precisely weighed salts in water. We also used solid NaF (Fluka, Germany) and such solutions as 1.5 mol dm^−3^ KSCN (Fluka, Germany), 0.1 wt.% 1-(2-pyridilazo)-2-naphtole (PAN) (Sigma-Aldrich, USA), 0.05 wt.% CuSO_4_ (Fluka, Germany), 0.02 mol dm^−3^ NaIO_3_ (Sigma-Aldrich, USA) in borate buffer solution with рН 9.2, citrate buffer solution with pH 4, 5 wt.% KI (Merck, Germany), 0.01 wt.% rhodamine 6G (Sigma-Aldrich, USA), 0.1 wt.% indigo carmine (Sigma-Aldrich, USA) in 50% ethanol (Merk, Germany), 1 wt.% pyrocathechin (Merk, Germany) in borate buffer solution with рН 11, 2 mol dm^−3^ K_2_CO_3_ (Merck, Germany), 3 wt.% H_2_O_2_ (Merck Millipore, Germany), 2 mol dm^−3^ H_2_SO_4_ (Merck, Germany) and 5 mol dm^−3^ HCl (Merck, Germany), and saturated solutions of K_2_S_2_O_8_ (Merck, Germany) and Na_2_СO_3_ (Merck, Germany).

The solutions of KSCN, CuSO_4_, KI, K_2_CO_3_, K_2_S_2_O_8_, and Na_2_СO_3_ were prepared by dissolving the precisely weighed salts in water. PAN and indigo carmine solutions were prepared by dissolving the precisely weighed substances in 96% and 50% ethanol, respectively. 3 wt.% H_2_O_2_ solution was prepared by diluting initial 30% solution H_2_O_2_ by distilled water. The solution of rhodamine 6G was prepared by dissolving the precisely weighed substance in water; the solutions of pyrocathechin and NaIO_3_ were prepared similarly by dissolving in borate buffer solutions with рН 11 and рН 9.2, respectively.

To prepare the borate buffer solution with pH 11, 49.9 mL of NaOH solution (0.1 mol dm^−3^) was poured into 100 mL volumetric flask and diluted by the solution of Na_2_B_4_O_7_ (0.05 mol dm^−3^). To prepare the borate buffer solution with pH 9.2, 98.1 mL of Na_2_B_4_O_7_ solution (0.05 mol dm^−3^) was poured into 100 mL volumetric flask and diluted by HCl solution (0.1 mol dm^−3^). To prepare a citrate buffer solution with pH 4, 56.0 mL of sodium citrate (0.1 mol dm^−3^) was poured into 100 mL volumetric flask and diluted with 0.1 mol dm^−3^ HCl solution. For preparation of the solution of sodium citrate, 21.014 g citric acid monohydrate and 200 mL of 0.1 mol dm^−3^ NaOH solution were placed into 1000 mL volumetric flask and the mixture was diluted by water.

As a sorbent, we used ether-based PUF of 22-3.0 type. PUF tablets with a diameter of 16 mm and of 0.025 g were stamped with metal punch from PUF plate with 5 mm thickness. After that, the tablets were purified by treating with 1 mol dm^−3^ H_2_SO_4_ (Merck, Germany) during 1 h and washed with water till obtaining neutral pH. Then the tablets were compressed using a glass stick and the rest of the solution was removed with filter paper and dried on air.

### 2.2. Apparatus and Software

The color *RGB* coordinates were measured by a portable reflectometer designed by the author of [12]. PUF tablet was placed on the reflectometer glass slide and pressed with snow-white Telfon plate. The average values of *R*, *G*, and *B* channels were obtained in the dialog window of the software developed by the manufacturer of the mentioned reflectometer. The reflectometer was calibrated using the etalon BaSO_4_ for diffusion reflectance spectrometry (DRS) as a standard of white color before *RGB* measuring.

The diffuse reflectance was measured using a special attachment to the spectrophotometer SF-2000 Bio (LOMO, Russia) for recording the diffuse reflectance spectra. The value of diffuse reflectance (*R*) was measured against a blank sample and counted in units of light absorption (*A*) which was used as an analytical signal.

The PUF tablet color in the test scales was measured by means of our earlier developed compact photometer “Phototest” [13, 14]. For the calibration curves, the EMF value was registered for the samples with known concentrations of the component to be determined and without them.

The test scales were scanned by an EpsonV350 Photo scanner in 24-bit color mode at 300 dpi resolution. The image processing was performed using Gimp 2.6.7 and Adobe Photoshop 8.0 software. For this purpose, the scanned image was selected and “Gauss dither” function at a radius of 30 pixels was used. Then the image center was found and “histogram” function was used for obtaining the average values of *L*, *A*, and *B* in the CIE *LAB* system [14]. To scan test papers, slide adapters were also used. The total color difference (Δ*E*) was found to be equal to (Δ*L* ^2^ + Δ*A* ^2^ + Δ*B* ^2^)^1/2^, where Δ*L*, Δ*A*, and Δ*B* are the changes of the color coordinates *L*, *A*, and *B*.

The results obtained with the reflectometer or the scanner were statistically processed and then the calibration curves were built for the readings of channels *R*, *G*, and *B* or the total color difference Δ*E* depending on the microimpurity concentrations.

To prepare test tools and test scales, we used the following materials and devices: filter paper, office paper, blister cells, enzyme immunoassay (EIA) plates, syringes, in particular, an insulin syringe for inject solution and a medical syringe without needle (5 mL for paper disc and 20 mL for PUF) for preconcentration, and a magnetic stirrer.

### 2.3. Making of PUF Test Scale for Determination of Co(II), Fe(III), Pd(II), and NO_2_^−^ (to Be Prepared in Advance)

The test scales were constructed according to a geometric progression (with a step of 1.5 or 2) or the Fibonacci series (with a step of 1.618). For the latter case, we used a new method proposed in [15] and based on the Fibonacci sequence (the rate of progression Φ = 1.618), in which each successive term in the series is the sum of the two previous terms (0, 1, 2, 3, 5, 8, 13, 21, 34, etc.) and, from the 5th term of the series and on, the rate of progression Φ = 1.618. The use of this sequence takes into account the advantages and disadvantages of using geometric and arithmetic progressions in test analysis.

The test scales for Co(II) and Fe(III) were prepared according to the techniques of complexing with thiocyanate [9]. For obtaining the colored analytical forms, the beaker of 50 mL was filled with the 20 mL of the solution containing the components to be determined in certain concentrations: 0.05–1.5 for Fe(III) and 0.1–1.5 for Co(II) (mg L^−1^), 0.2 mL for Н_2_SO_4_, and 5 mL for KCNS. In the case of Co(II), 0.1 g of NaF was introduced additionally into the beaker. In each beaker a prepurified PUF tablet was put after removing air bubbles with a glass stick. The beaker's content was stirred during 15 min by means of magnetic stirrer or by hand. Then the tablet is removed from the solution and squeezed with a glass stick; the rest of the solution was removed with filter paper and left to dry in air.

To prepare the test scale for NO_2_^−^, the reaction of diazotization directly of PUF was used. Each prepurified PUF tablet was put in a 50 mL beaker filled with 20 mL of the solution of a certain concentration of NO_2_^−^ (0.1–4 mg L^−1^) and 5 mL of HCl and impregnated for 15 minutes by stirring by hand or using a magnetic stirrer. Then the tablet was removed from the solution and squeezed with a glass stick; the rest of the solution was removed with filter paper and dried in air.

For preparing the test scales for Pd(II) determination, 3 mL of KI solution, 1 mL of rhodamine 6G solution, and certain amounts of Pd(II) solution (0.04–1 mg L^−1^) were added to 2.5 mL of citrate buffer and diluted to 25 mL by distilled water. The optimal amount of rhodamine 6G (1 mL of 0.01% solution) necessary for sorption extraction of Pd(II) on PUF in static mode was found. The obtained solution was moved to the flask with prepurified PUF tablet for carrying out sorption of colored complexes for 15 min. Then the PUF tablet was removed from the solution and squeezed with a glass stick; the rest of the solution was removed with filter paper and dried in air. The color coordinates of the PUF tablets were measured by a portable digital reflectometer.

### 2.4. Making of Paper Test Scale for Co(II) Determination (to Be Prepared in Advance)

The filter paper circles with a diameter of 10 mm were placed on glass slides, impregnated with 10 *μ*L of PAN solution, and dried in air. Then 10 *μ*L of the solution containing 0.1–1 mg L^−1^ of Co(II) was added with the paper and dried in air again.

### 2.5. Making of Sandwich Test Tool for Co(II) Determination (to Be Prepared in Advance)

*Variant 1*. Filter paper was cut into 40 × 40 mm squares. One part of the obtained paper samples was impregnated with 50 *μ*L of the dye (pyrocathechin solution in borate buffer with pH 11) and dried in air. The other part was impregnated with 50 *μ*L of the oxidizer (H_2_O_2_ solution) and dried in air. After that, the prepared dry paper samples (with the dye and oxidizing agent) were packed into Mylar film in the form of two-layer sandwich.

*Variant 2*. Filter paper was cut into 17 × 17 mm squares, impregnated with 50 *μ*L of K_2_CO_3_ solution, dried in air, and impregnated with 20 *μ*L of indigo carmine solution and dried again. The other paper pieces (17 × 17) were impregnated with 20 *μ*L of K_2_S_2_O_8_ and also dried. After that, two prepared dry paper pieces (with the dye and oxidizing agent) were packed into Mylar film in the form of two-layer sandwich.

In both variants, the side of the sandwich with oxidizer had a small hole (2-3 mm in diameter) for injection of the solution to be analyzed.

The calibration graphs were constructed in advance using the solutions containing Co(II) in the amount of 0.005–1 mg L^−1^ (with pyrocathechin) or 0.5–10 mg L^−1^ (with indigo carmine).

### 2.6. Preparation of Blister Test Tool for Rh(III) Determination

The tool* was prepared online*. Blister cells or transparent EIA plates were filled with 50 *μ*L of sodium periodate solution in borate buffer, with 50 *μ*L of CuSO_4_ solution, and dried. Then all the cells were sealed together by adhesive tape for their protection and then used to construct the scale in the outdoor conditions. To build the test scale, we injected 50 *μ*L of the solution containing different amounts (0.01–0.8 mg L^−1^) of Rh(III) into each cell straight through a protective adhesive tape by means of an insulin syringe.

To simulate the color test scale* prepared in advance*, the colored liquid-based scale in blister cells or transparent EIA plates was scanned by flatbed scanners. Images were stored in JPEG format with minimal compression. The color coordinates *R*, *G*, and *B* of the scanned images obtained using a graphical editor Gimp were averaged based on a large number of parallel experiments (*n* = 10–20). Afterwards, the averaged color scale was built and printed on the office paper.

## 3. Results and Discussion

### 3.1. Portable Devices

Colorimetry as a physical method of chemical analysis based on determination of the concentration of a substance by absorption of light by solutions. In contrast to the physical method of colorimetry,* digital image colorimetry* is based on measurements of the color characteristics (color coordinates) of colored components or their images, for example, by means of three-color digital colorimeters. These colorimeters use the *RGB* system based on additive mixing of three basic colors (red, green, and blue). Recently, in order to digitize color images, the use of devices such as digital cameras, web-cameras, and flatbed scanners in combination with image processing software (by means of image editors) has been reported in the literature. The cheapness and availability of such equipment as well as the quality of the analysis at the level of industrial colorimeters make these devices very attractive for rapid analysis by digital image colorimetry.

Scientists from the Kharkiv National University of Radioelectronics [12] have designed an inexpensive portable digital reflectometer supplied with the software for instrumental rapid testing of a variety of solid samples (0.1% accuracy), including powders of sorbents (silica gel, zeolite, aluminum oxide, etc.), disks of sorbents, polyurethane foams, indicator papers, and polymer films. The Institute for Single Crystals has developed a simpler photometric device—a pocket photometer “Phototest” (1% accuracy) for testing solid semitransparent samples [13]. The device measures the EMF caused by the light passing through a colored sample that depends on the coloration intensity.

The authors compared the properties of the mentioned photometer, reflectometer, and the scanner by means of detecting the model impurities of Co(II) and Fe(III) as thiocyanate complexes in water and NO_2_^−^ by the diazotization reaction on PUF [14]. The said analytical forms were chosen due to their colors (blue, red, and yellow, resp.) covering the whole of the visible spectrum.  Table 1 summarizes the results of identification of the abovementioned compounds sorbed on PUF tablets by using various portable devices. The obtained results were subjected to statistical processing according to recommendations [16, 17]. The limit of quantification (LOQ) and the relative standard deviation (RSD) necessary for comparing the analytical capabilities of these devices were estimated. As a rule, LOQ is estimated from the blank using 6*σ* criterion (in contrast to the limit of detection (LOD) estimated using 3*σ* criterion). This method requires the presence of a noticeable background, from which the signal (after replicate measurements) is to be processed to estimate LOD. If the blank is too small, the result corresponding to minimum concentration can be processed instead of it. Sometimes, for various reasons it is impossible to prepare the blank; then the limit of detection cannot be evaluated in such a way. According to [16], the use of the data on fluctuations in the blank gives an ambiguous assessment of LOQ or LOD. Therefore, as shown in [16], the most correct and easily implemented evaluation of these characteristics can be finding such a value of the analyte concentration that can be determined with a predetermined error. Most often, for LOD evaluation, RSD equal to 0.5 is set (since the concentration found with higher RSD becomes a statistical zero), and for the LOQ assessment RSD equal to 0.3 is set. In the latter case, the concentration found is still far from a statistical zero. Sometimes for strict measurements, RSD equal to 0.2 is set. In practice for finding LOQ, it is convenient to use an approach based on experimentally constructed concentration dependence of RSD. In fact, you can practically always realize an experiment whose results will be obtained with both small and large errors. So, this dependence within the entire operating range of concentrations will be strict. For this dependence, one can find the LOQ value corresponding to RSD = 0.3.

As seen from Table 1, the most precise results were obtained by the reflectometer. Although the measurement error of “Phototest” photometer is higher than that of the reflectometer, it is 2–5 times lower than the visual test analysis with RSD of 0.25–0.3.

**Figure 1:**
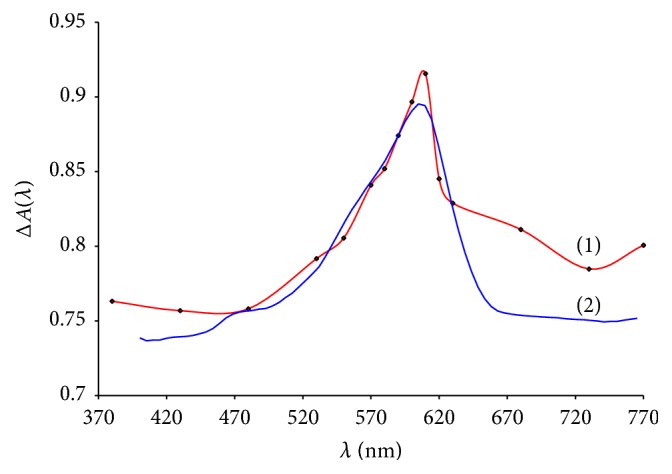
Absorption (1) spectrum obtained on “Phototest” with filter kit and diffusion reflectance (2) spectrum obtained on “SF-2000 Bio” with diffusion reflectance add-on kit.

The improvement of the photometer “Phototest” by using an additional set of film color filters allowed converting it into a simple photocolorimeter [13, 14].  Figure 1 demonstrates the examples of the measurement of the absorption spectrum for the colored PUF tablet with the said device in comparison with the results obtained by the most commonly used DRS method. It is seen that the spectra are identical, so the use of this portable device for express analysis is promising.

**Figure 2:**
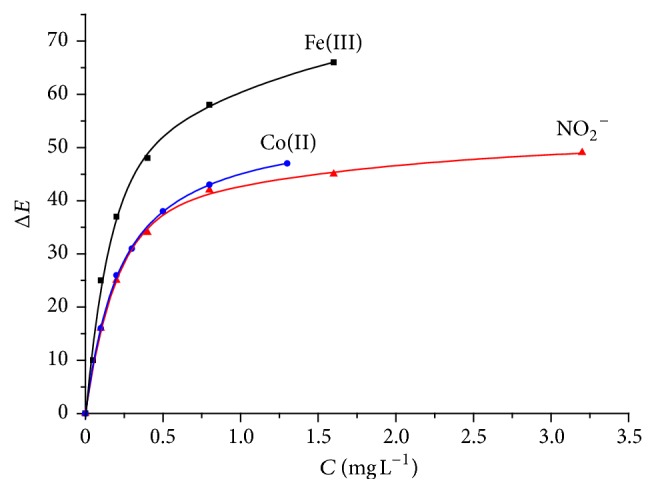
Concentration dependence of the total color difference (Δ*E*) calculated from the scanned images of the color scales on PUF tablets for the colored components: Co(II), Fe(III), and NO_2_^−^.

For digitizing color images in the field conditions, a commercial flatbed scanner can be used. Registration of analytical signals was found to be more convenient using the device in the form of test paper packed in a slide adapter. This simplifies the placement of several test tools simultaneously on a flatbed scanner and accelerates scanning and image processing. The correlation between the total color difference (Δ*E*) obtained from the results of scanning and the measured concentration of model colored component (as mentioned above for Co(II), Fe(III), and NO_2_^−^) is shown in Figure 2. Note that Δ*E* serves as a universal criterion for choosing the step of color scale in visual test analysis.

### 3.2. Carriers for Co(II)

**Figure 3:**
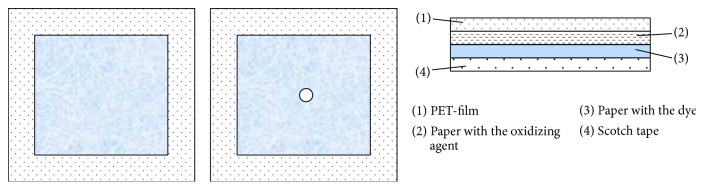
Test tool for kinetic determination of Co(II) based on double-layer paper “sandwich.”

A proper choice of solid carrier when creating a test tool is of great importance for a successful test analysis. It was found that, for the kinetic determination of Co(II) which catalyzes the reaction of oxidation of pyrocatechin [6] or indigo carmine [5], the most suitable test tool was a double-layer paper “sandwich” [18] soaked with appropriate reagents and packed into a protective polymer film. As an analytical signal, coloration was used, which in the presence of Co(II) appeared (variant 1, brown-pink coloration) or disappeared (variant 2, blue coloration) in time. The sandwiches consisting of two layers of paper impregnated with reagents and packed in a polymer film were prepared for kinetic visual determination of Co(II) in two variants. The best oxidant in the 1st variant appeared to be H_2_O_2_ only. Application of any other oxidant, for example, K_2_S_2_O_8_, may result in side coloration that distorts the analytical signal. It was established that placement of the dye in a single layer and oxidant together with buffer solution in another layer was optimal. Such a separation of the reagents is necessary for avoiding their mutual influence during storage. In the 2nd variant, the indicator layer contained indigo carmine and Na_2_CO_3_, whereas the oxidizing layer contained K_2_S_2_O_8_ as the most suitable oxidant (Figure 3). On the upper (oxidant) side, there was a hole in the film for injection of the studied solution. The said test tools can be made in less than 1 h. The test tools should be stored in the dark.

**Figure 4:**
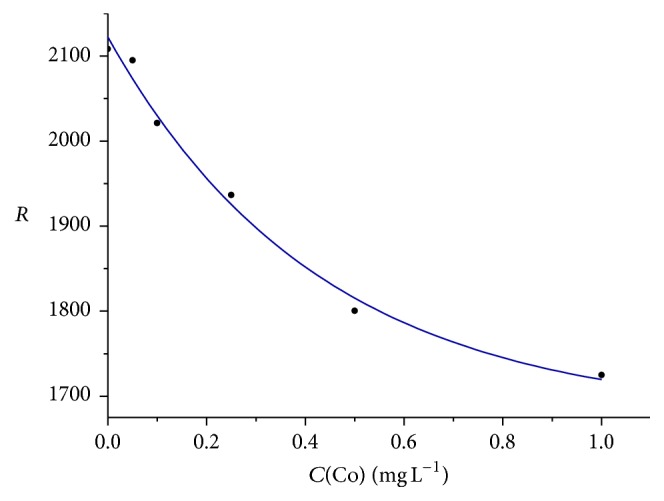
Concentration dependence of the color coordinate *R* of the colored spots measured by digital portable reflectometer for determination of Co(II) by the reaction of pyrocatechin oxidation.

It has been shown that in the 1st variant the color coordinates of colored spots can be measured in the field conditions with a scanner or digital portable reflectometer designed at the Kharkiv National University of Radioelectronics [12]. In the case of the scanner, at several paper sandwiches were packed each to its own slide adapter and positioned on the surface of the scanner. Then the analyzed solution was spread on the sandwiches and after a fixed time the color coordinates were determined. In the case of the reflectometer, the said treatment is realized only for one sample to be analyzed. Unknown concentration of Co(II) in water samples was determined by the calibration graph previously constructed in the coordinates: channel *R*-*C* (Figure 4).

**Figure 5:**
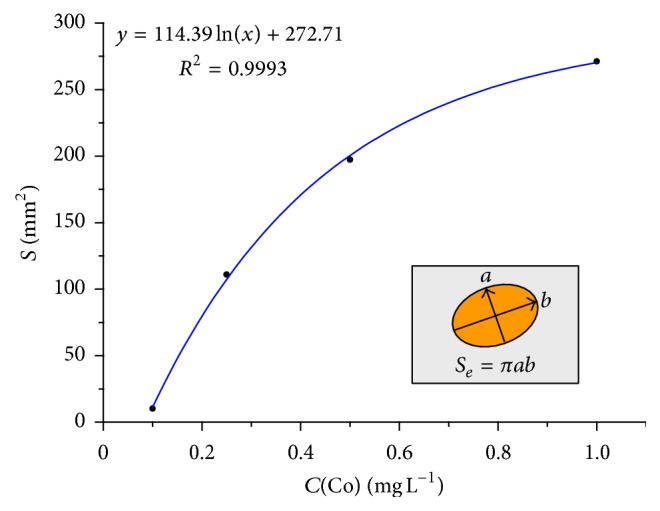
Concentration dependence of the colored spot area for Co(II) determination by the reaction of pyrocatechin oxidation.

It is interesting to note that the test allows determining Co(II) with pyrocatechin using the area (*S*) of the spot (which has an oval shape) as the analytical signal instead of the color of the spot. This area can be easily calculated with the help of a simple ruler: just measuring the big (*a*) and the small (*b*) axis of the oval and calculating *S* = *πab*. Prior to the analysis, the calibration curve (within the range of Co(II) concentrations of 0.1–1 mg L^−1^) should be constructed in the coordinates *S*-*C* (Figure 5). The unknown Co(II) concentration in a water sample is identified by the calibration curve.

To enhance sensitivity, as an alternative to catalytic reactions, more reliable reactions of simple complex formation with analytical ligands can be used in the test. For example, 1-(2-*pyridylazo*)-2-*naphthol* (PAN) fixed on the silica gel [19] proved to be the most promising test tool for visual detection of Co(II) in natural water in the form of its blue complex. By means of special experiments, it has been shown that the results of this analysis, unlike those catalytic reactions, do not depend on the degree of mineralization and hardness of natural waters.

In order to avoid any subjective factors inherent in visual tests, detection of Co(II) in the form of* thiocyanate *complexes on PUF tablets [9] involved digitalization and processing of the obtained results. The color scales were built for Co(II) concentration range of 0.1–3.2 mg L^−1^; the scale step is equal to 1.5, 2 as well as 1.618 (Fibonacci series) [15]. The coordinates of the colored tablets were measured by means of a flatbed scanner or portable reflectometer or by the same tablets for light absorption by “Phototest” spectrometer [13]. When logarithmically linearized [14], the calibration graph was linear within Co(II) concentration range of 0.1–1.3 mg L^−1^.

### 3.3. Carrier for Rh(III)

While choosing the carrier for the tests on Rh(III) by means of the reaction of catalytic oxidation of Cu(II) by periodate in the presence of Rh(III) [6], the reagent solutions were placed in the carrier and the appearance of yellow color was observed. The following solid carriers were studied: filter paper and office paper, silica gel C60 (used in chromatography), *α*- and *γ*-oxide aluminum, zeolite, xerogel, magnesium oxide, cotton and synthetic cloth, PUF, films of water soluble and insoluble polymers, and polyethylene terephthalate (PET) blister cells. It was found that the best option carriers were the ones of only PET due to their smooth surface.

EIA plates and PET blister cells proved to be the most successful and convenient in practice. To make a test, the chemical solutions were placed into blister cells or EIA plate and left to dry at room temperature. Then the cells were sealed by adhesive tape. An insulin syringe was used to inject the specimen solution beneath the protective tape, and the reaction commencement time was noted. The cells were placed on a white background, and their coloration was compared with the blank test blister or EIA plate (without Rh(III)) via a fixed time interval. To eliminate subjective factors and simplify the outdoor analysis, imitation colored scales were prepared on the test paper. For this purpose, many liquid scales in the blister sells or transparent EIA plates were scanned, and the color coordinates *R*, *G*, and *B* were obtained; then the average scale was printed on the office paper. In the process of analysis, the coloration in the cell with the studied solution through a fixed time after the addition of reagents was compared with the paper scale printed in advance. It was found, unfortunately, that the rising salinity of the analyzed water negatively affected the color appearance time.

### 3.4. Carrier for Pd(II)

Another example of the mentioned replacement of catalytic reactions by reactions of complex formation (similar to Co(II) with thiocyanate) is the formation of colored ternary complex of Pd(II) with said reagent and iodide in weakly acidic medium [8].

**Figure 6:**
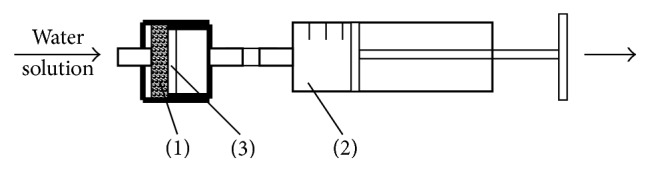
Pocket concentrator with special cell (1) based on medical syringe (2) for the test tool in the form of paper or PUF disc (3).

Although the techniques based on simple complexation are less sensitive, they are simpler and more reliable than any catalytic techniques. That is why their use is expedient when one needs to identify relatively high concentrations of the abovementioned elements, for example, in the relevant process solutions or in natural water during environmental disaster. However, the sensitivity of the discussed complexing methods applicable for Co(II) and Pd(II) detection on PUF, as well as for Co(II) on paper with PAN, can be considerably enhanced by preconcentration of the impurities [9]. For this purpose, the authors applied a pocket concentrator based on medical syringe in which the needle was replaced by a special cell containing the test tool in the form of paper or PUF tablet (Figure 6). Preconcentration was accomplished by repeated pumping of the solution containing a colored complex through the syringe. This combination allowed reducing the detection limit by 2 orders of magnitude, thus making this version comparable with the kinetic determination. Naturally, this device can be used in the field conditions just before the testing analysis.

Summarized Table 2 describes the metrological properties of the visual and instrumental test methods of rapid outdoor testing of natural water with the help of the tools developed by the authors. The obtained results were statistically processed in accordance with recommendations [16, 17]. For the semiquantitative determination, LOD was set as the content of the required element corresponding to the criterion 3*σ*. The evaluation of LOQ for the quantitative determination was carried out at RSD = 0.3 or at 6*σ* of the blank. If such criteria could not be applied, the first point on the calibration graph/scale served as LOQ.

To verify the trueness of results, the found content of the required elements in the solution was compared with their contents entered. The differences between them were not significant.

The effect of foreign cations which may be present in natural water was studied. At the determination of Co(II), Rh(III), or Pd(II) in water, simultaneous presence of Mg(II), Ca(II), Fe(III), Cu(II), and Al(III) does not influence the obtained results.

### 3.5. Procedure for Co(II), Pd(II), and Rh(III) as well as Fe(III) and NO_2_^−^ Tests

The analysis duration depends on the concentration of the analyte (in the case of kinetic reactions) and is determined by the time required for impregnating the carrier (in our case or paper or PUF disc) with the solution to be analyzed.

*NO*_2_^−^. For the analysis, the operator impregnates a PUF tablet with the solution to be analyzed as described in Section 2. After drying by means of filter paper, the color of the tablet is compared with the previously prepared color scale. The analysis time is 15 minutes.

*Fe(III) or Co(II)*. For the analysis, the operator prepares a PUF tablet on the base of thiocyanate and the solution to be analyzed as described in Section 2. Then the color of the tablet is compared with the previously prepared color scale. The analysis time is 15 minutes.

*Co(II)*. For the analysis, the operator injects 20 *μ*L of the analyzed solution into the injection hole of the “sandwich” by means of an insulin syringe (see Section 2). The onset of coloration (variant 1, with pyrocatechin) or discoloration (variant 2, with indigo carmine) time is fixed. The analysis duration depends on the content of Co(II). Unknown concentration of Co(II) in water samples is determined visually or instrumentally. In particular, in the case of* visual test* for Co(II) with pyrocatechin (variant 1), the color of the spot on the “sandwich” to be analyzed is compared with the previously prepared color scale after 1 or 5 min of the reaction onset. The scanner or reflectometer is used in the case of* instrumental test*. The color coordinates are determined after 1 or 5 min of the reaction onset and unknown concentration of Co(II) is determined by the calibration graph previously constructed in the coordinates channel *R*-*C*. In the case of* measuring area*, the big and small axes of the oval spot are measured by a ruler and the area is calculated. The Co(II) concentration in water samples is identified by the calibration graph previously constructed in the coordinates *S*-*C*.

For the analysis, the operator prepares paper discs on the base of PAN and the solution to be analyzed as described in Section 2. Then the color of the tablet is compared with the preliminarily prepared color scale. The analysis duration is 15 minutes. If necessary, one can carry out preconcentration of the colored complex by means of pumping the solution using a pocket concentrator (a medical syringe with the special cell).

*Pd(II)*. For the analysis, the operator prepares a PUF tablet on the base of the solution to be analyzed as described in Section 2. Then the color of the tablet is compared with the previously prepared color scale. The analysis duration is 20 minutes. If necessary, one can carry out preconcentration of the colored complex by pumping the solution ten times through a pocket concentrator (a medical syringe with a special cell).

*Rh(III)*. For the analysis, the operator injects 50 *μ*L of the solution containing certain amounts (0.01–0.80 mg L^−1^) of Rh(III) into preliminarily prepared blister cells (see Section 2) straight through protective adhesive tape by an insulin syringe. Simultaneously another blister cell is filled with the solution to be analyzed. The time of the appearance of yellow color of the solution contained in a blister cell serves as analytical signal. Then the time of the appearance of the color in the cell with the analyzed solution is compared with that of the color in the cells with Rh(III). The duration of the analysis is 20–30 minutes depending on the content of Rh(III).

In the case of using a simulated colored scale printed in advance, the coloration in the cell containing the solution to be analyzed was compared with that scale within 30 minutes after the addition of the reagents.

## 4. Conclusion

It is proposed to use visual and instrumental detection of Co(II), Pd(II), and Rh(III) in the form of colored compounds for monitoring natural water in the field conditions. As the test tools, the following ones were fabricated: PUF tablets with the sorbed colored complexes, the two-layer paper “sandwich” impregnated by the reagents and placed in slide adapter, and PET blister with dried reagents. The use of portable reflectometer, pocket photometer, or scanner-technology as well as pocket concentrator allows estimating the color coordinates and improving the accuracy and sensitivity of the test analysis outside the laboratory. It is established that the sensitivity and selectivity of the visual tests are comparable with the sorption-spectrophotometric technique using the simple portable pocket photometer “Phototest” developed by the authors. This approach allows eliminating a negative effect of subjective factors on the analysis results and can be successfully applied for environmental express-control of natural water.

## Figures and Tables

**Table 1:** The comparison of test results using various portable equipment^**∗**^.

Equipment	Model impurity	Concentration range, mg L^−1^	LOQ, mg L^−1^	RSD (for median range)
Reflectometer	Со(II)	0.1–1.5	0.012^*∗∗*^	0.01
	Fe(III)	0.1–1.5	0.08^*∗∗*^	0.01
	NO_2_^−^	0.3–2.4	0.016^*∗∗*^	0.05

Photometer “Phototest”	Со(II)	0.1–1.3	0.08^*∗∗∗*^	0.06
	Fe(III)	0.05–0.4	0.14^*∗∗∗*^	0.17
	NO_2_^−^	0.3–3.9	0.38^*∗∗∗*^	0.15

Scanner	Со(II)	0.1–1.3	0.05^*∗∗∗*^	0.06
	Fe(III)	0.05–1.5	0.025^*∗∗∗*^	0.13
	NO_2_^−^	0.1–3.2	0.02^*∗∗∗*^	0.12

^*∗*^RSD is relative standard deviation expressed as a decimal fraction; LOQ is limit of quantification (sample volume *n* = 5).

^*∗∗*^LOQ under 6*σ* of the blank.

^*∗∗∗*^LOQ under RSD = 0.3.

**Table 2:** The metrological characteristics of the developed tests for Co(II), Rh(III), and Pd(II).

Impurity	Analytical reaction/test tool/	Indication way	Concentration range, mg L^−1^	LOD or LOQ, mg L^−1^	RSD in decimal fraction (for median range)
Co(II)	Indigocarmine oxidation by H_2_O_2_ in the presence of Co(II)/sandwich/	Visual	0.5–10	0.5^*∗*^	—
	Pyrocathechin oxidation in the presence of Co(II)/sandwich/	Visual	0.01–1.0	0.01^*∗*^	—
		Instrumental (*RGB*)	0.005–0.1	0.005^*∗∗*^ 6*σ*	0.06
		Instrumental (square of the spot)	0.01–1.0	0.01^*∗∗*^ the first point	0.12
	Formation of color complex with PAN/paper/	Visual	0.25–10	0.25^*∗*^; 0.25^*∗∗*^ the first point	—
			0.002–0.1 (preconcentration)	0.002^*∗∗*^ (preconcentration)	
		Instrumental	0.1–10; 0.001–0.1 (preconcentration)	0.05^*∗∗*^ 6*σ*; 0.001^*∗∗*^ (preconcentration) the first point	0.05
	Formation of color complex with thiocyanate/PUF/	Visual	0.1–1.5	0.02^*∗*^	—
		Instrumental	0.01–1.5	0.01^*∗∗*^ RSD = 0.3	0.01

Rh(III)	Oxidation of Cu(II) by periodate in the presence of Co(II)/blisters/	Visual	0.01–0.8	0.005^*∗*^	—
		Instrumental	0.01–0.8	0.005^*∗∗*^ 6*σ*; 0.01^*∗∗*^ RSD = 0.3, the first point	0.1

Pd(II)	Formation of color complex with rhodamine 6G/PUF/	Visual	0.04–0.8	0.04^*∗*^	—
		Instrumental	0.04–0.8	0.06^*∗∗*^ 6*σ*; 0.08^*∗∗*^ RSD = 0.3, 0.04^*∗∗*^ the first point	0.1
		Visual, instrumental	0.004–0.08 (preconcentration)	0.004^*∗∗*^ (preconcentration) the first point; 0.008^*∗∗*^ (preconcentration) RSD = 0.3	0.1

Sample volume *n* = 10; ^*∗*^LOD; ^*∗∗*^LOQ.
